# How Irrigants Affect Mineral Trioxide Aggregate (MTA) Sealing in Furcal Perforations: An In Vitro Study

**DOI:** 10.7759/cureus.70690

**Published:** 2024-10-02

**Authors:** Ghaffar Dayyoub, Mouhammad Al-Tayyan, Yasser Alsayed Tolibah, Hassan Achour

**Affiliations:** 1 Endodontics, Damascus University, Damascus, SYR; 2 Pediatric Dentistry, Damascus University, Damascus, SYR

**Keywords:** dye penetration, furcal perforations, irrigants, marginal leakage, mta proroot

## Abstract

Objective

This study aimed to evaluate the effect of 5.25% sodium hypochlorite (NaOCl) and 2% chlorhexidine (CHX) on ProRoot mineral trioxide aggregate (MTA) used for treating furcal perforations, compared to no solution application, using the dye penetration (DP) method.

Materials and methods

The study included 36 intact mandibular molars in the furcal area (FA) with well-spaced roots. Using dental operating microscopes, perforations were created at the FA of the molars with a 1.2 mm diameter. Subsequently, ProRoot MTA (Dentsply, Germany) was applied to seal the perforations. After 24 hours, the samples were randomly assigned to three groups: Group A (n = 12), irrigated with 5.25% NaOCl; Group B (n = 12), irrigated with 2% CHX; and Group C (n = 12), without irrigation (control group). Specimens' pulp chambers were immersed in 2% methylene blue for 24 hours. Buccal-lingual sections were performed, and the depth of marginal dye leakage was assessed using a stereomicroscope at 20x magnification. Data analysis employed the one-way analysis of variance (ANOVA) test and Bonferroni post-hoc test (α = 0.05).

Results

The study revealed significant differences in DP values between groups (p-value < 0.01). DP values in the control group (no irrigation) were lower than both NaOCl (p < 0.05) and CHX (p < 0.01) groups. Furthermore, DP values in the CHX group were higher than those in the NaOCl group (p < 0.01), suggesting the order: no irrigation < NaOCl < CHX (p < 0.05).

Conclusions

The sealing ability of MTA was compromised when irrigated with 2% CHX and 5.25% NaOCl. CHX significantly impacted the marginal leakage and sealing ability of MTA. Care should be taken when resuming endodontic treatment after the application of MTA in cases of furcal perforations as the use of CHX and NaOCl may affect the marginal leakage of MTA.

## Introduction

Perforation, an adverse occurrence in dental practice, can manifest in various ways, affecting the pulp chamber floor or root [1]. Accidental furcal perforation, an iatrogenic complication, may arise during restorative or root canal procedures [2]. Multirooted teeth, particularly mandibular molars, are predominantly affected, with 54.31% of occurrences observed in this area [3]. Delaying furcal perforation repair may lead to periodontal breakdown due to microbial infiltration. Timely intervention to repair the perforation is crucial to prevent potential tissue damage, especially before initiating root canal therapy [2]. The prognosis of perforation depends on the contamination time and the size of the perforation location. Additionally, the choice of sealing material significantly impacts treatment outcomes [4].

Mineral trioxide aggregate (MTA), a first-generation calcium silicate cement, has gained popularity in endodontic and pediatric dentistry due to its regenerative characteristics and improved physical properties. MTA promotes cementum regeneration, exhibits biocompatibility with periradicular tissues, sets in a moist environment, acts as a barrier against bacterial ingress, induces matrix production for cementum synthesis, facilitates hemostasis, and has low solubility after setting. Furthermore, MTA can induce mineralized tissue formation [5]. When used for perforation repair, MTA demonstrates superior sealing ability compared to amalgam and intermediate restorative material (IRM) by stimulating cement cells to produce a matrix for cementum formation [6]. MTA has shown favorable outcomes in various clinical scenarios, including furcal perforation, lateral radicular issues, apicectomy, radicular resorption, direct pulpal capping, and apexification cases [1].

Following furcal perforation repair, a thorough disinfection of the root canal system is necessary using a spectrum of irrigating solutions. However, little information exists on the effects of these solutions on the solidity and sealing properties of repaired furcal perforations [2].

The widely acknowledged antimicrobial irrigant is a 5.25% sodium hypochlorite solution (NaOCl), known for its ability to dissolve proteins indiscriminately, thereby affecting the physical properties of dentin [7]. A widely used irrigant in endodontic practice is 2% chlorhexidine (CHX), which is used as one of the final irrigation solutions for the necrotic root canals in the context of single visit treatment, renowned for its broad-spectrum antimicrobial efficacy against both Gram-positive and Gram-negative bacteria, minimal cytotoxicity, and advantageous substantivity [8,9].

There is a lack of literature on the impact of irrigants on the marginal leakage of MTA used for furcal perforation repair. While some studies have assessed the push-out bond strength of hardened MTA that exposed to different irrigant solutions [9,10], a previous study compared the effect of NaOCl and EDTA on the sealing ability of MTA and found that both solutions have a negative effect on the sealing ability of hardened MTA in its initial setting stages [11]. To the best of the researchers' knowledge, no study has addressed the impact of 2% CHX on the marginal seal of MTA. Therefore, the primary aim of this study was to investigate whether irrigation influences the sealing properties of hardened MTA. The secondary objective was to compare the marginal leakage effects of NaOCl and CHX on hardened MTA in furcal perforation repair.

The first null hypothesis posits that the tested irrigation solution does not affect the sealing properties of the repair materials, as evaluated through dye penetration (DP). The second null hypothesis suggests that there is no significant difference in the effect of various irrigants on the repair materials, as assessed by DP. This article was previously posted on the research square as a preprint on July 03, 2024 [12]. 

## Materials and methods

This study was approved by the Ethics Committee of Damascus University, Faculty of Dentistry (IRB No. 157 in 27/09/2021). This article reports the findings of an experimental, in vitro portion of the study. This study was conducted in Department of Endodontics, Faculty of Dentistry, Damascus University from January 15, 2023 to June 15, 2024. 

Sample size calculation

Drawing from the findings of a previous study [13], the sample size for this current study was determined utilizing G* Power 3.1.9.4 (Heinrich-Heine-Universität, Düsseldorf, Germany). In the analysis of variance (ANOVA), sample sizes of 12 were derived for each of the three groups, resulting in a total sample of 36 subjects. This configuration yields an effect size (f) of 0.60 (which was calculated according to the changing in the DP value for MTA used to repair large furcation perforations), the maximum and 85% power to discern disparities at a significance level of 0.05.

Sample selection

The study used 36 recently extracted permanent human mandibular molars. The included molars were extracted for orthodontic or periodontal reasons. The inclusion criteria consisted of molars with well-developed unmerged roots. The exclusion criteria were molars with carries, cracked molars, immature molars, molars with internal or external resorption, or or those with previous root canal treatment. The included molars in the study were examined using a 2.5 magnification lens (Carson handheld, Ronkonkoma, New York, USA) and periapical radiographs to identify the included molars. All extracted molars were disinfected and stored in a 0.5 chloramine-T at room temperature until use [14].

Specimen preparation

A high-speed 12-diamond ball bur (Dentsply Sirona, Ballaigues, Switzerland) was used to gain an initial entry and prepare an endodontic access cavity, followed by Endo-z bur (Dentsply Sirona, Ballaigues, Switzerland) that was used to remove the entirely pulp chamber roof and to finish the cavity walls. The teeth were then rinsed with water, and the contents of the pulp chamber and root canals were removed.

The molars were placed within an acrylic base 3 mm away from the buccal junction to make a space under the furcal area (FA) for the placement of a matrix.

**Figure 1:**
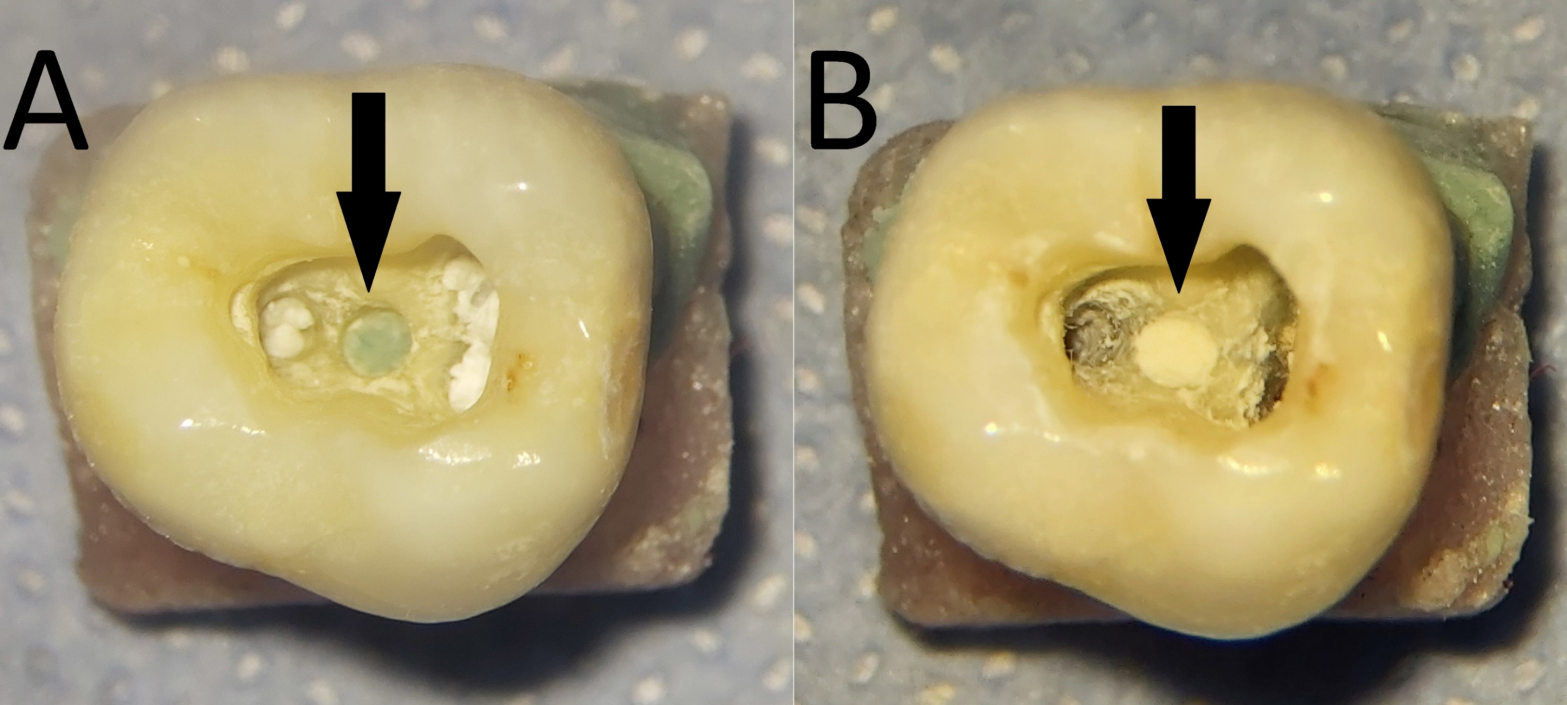
Sample preparations: (A) A simulated furcal perforation in the center of the furcation area and (B) the furcal perforation repairing with MTA MTA: Mineral trioxide aggregate

The Dental Operating Microscopes (DOM) (Suzhou Semorr Medical Tech Co., Ltd., Foshan, China) were used to make the simulated perforations in the center of the pulp chamber floor using a 1.2 mm round bur (SF-R11, Dianfong, Guangdong, China) in a slow-speed handpiece (Being Foshan Medical Equipment Co. Ltd., Guangdong, China), which set the width of the perforation as 1.2 mm (Figure 1A). The perforations length depended on the dentin and cementum thickness. 

Perforation repair

**Figure 2:**
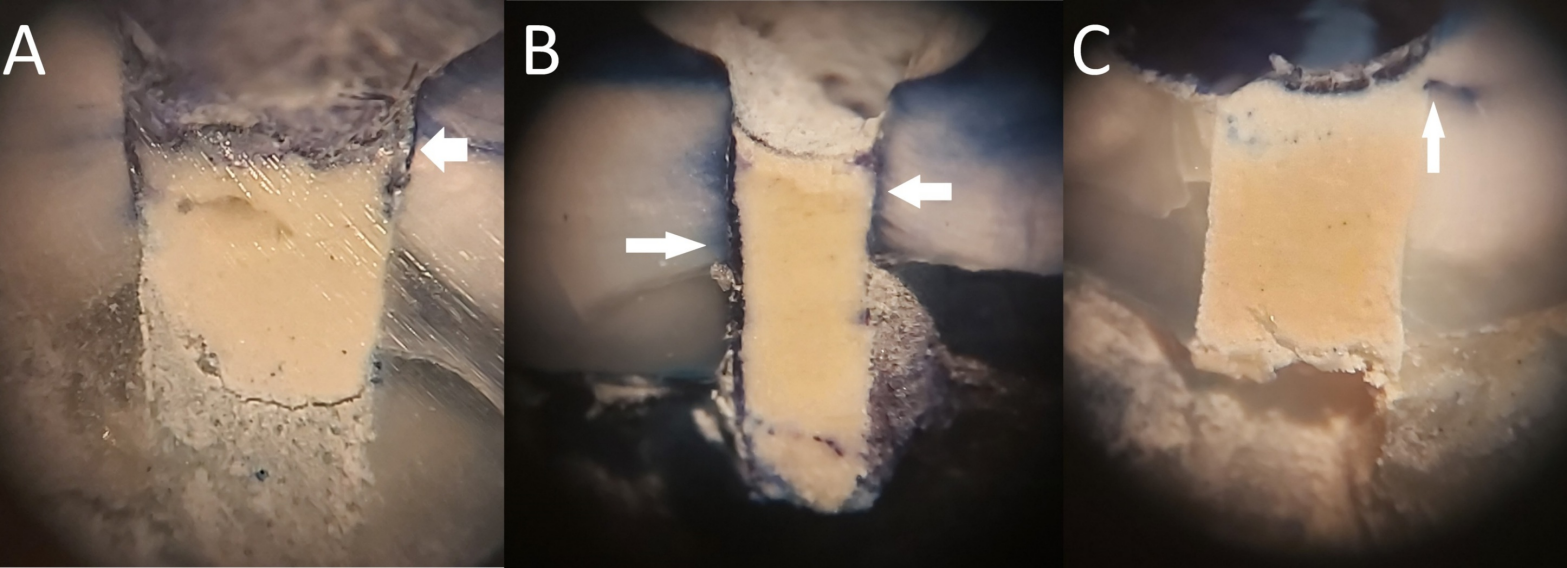
Dye penetration appeared in groups. (A) Group A, (B) group B, and (C) group C

A gelatine sponge (Gelatamp Roeko, Colten Whaedent, Switzerland) was condensed with a dental plugger (Fanta Dental Materials Co., Ltd., Shanghai, China) below the FA as a matrix, and the canal orifice was closed using cotton balls. Subsequently, the MTA (ProRoot MTA, Dentsply, Germany) was mixed according to the manufacturer's recommendations, and it was gently placed into the perforation site by an experienced endodontist (G.D.) using a carrier (D&P, Forgeman, Pakistan). The material was then condensed with a dental plugger under a DOM (Suzhou Semorr Medical Tech Co., Ltd., Foshan, China) (Figure 2A). The cotton balls were removed, and any excess material was carefully removed from the surface of the samples using a scalpel. Subsequently, sticky wax materials were placed in each canal orifice, and a saline-moistened cotton pellet was positioned over the MTA according to manufacturer instructions to ensure good setting of the MTA. The perforation site was sealed with a temporary filling. All samples were stored in 100% humidity at 37°C for 24 hours.

Irrigation

After incubation, numbers were assigned to the samples and randomly distributed among the three groups using the randomization website: www.Randomization.org. The samples were divided into three groups according to the applied irrigants: Group A (n = 12) were irrigated later with 10 ml of 5.25% NaOCl (Al Fares, Damascus, Syria) for 10 minutes [2]​, Group B (n = 12) were irrigated later with 5 ml of 2% chlorhexidine-digluconate (CHX) (Al Fares, Damascus, Syria) for three minutes [15]​, and Group C (n = 12) with no irrigant (control group) [16]. 

Irrigants were applied gently with a 27G needle (Sybron Endo, Orange, CA, USA). Afterward, the irrigants were withdrawn and washed with saline serum. This was done to control the working time and prevent the prolonged effect of the irrigant solution on the MTA. The pulp chamber and the canals’ orifice were dried with cotton balls and coated with nail varnish except the perforation site.

DP test

The pulp chambers of the molars were immersed in a 2% methylene blue solution (Terry's Polychrome Methylene Blue 2% Aqueous, Polysciences, Inc., Warrington, United States) for 24 hours. Following this period, all samples underwent cleaning under running water to eliminate the methylene blue dye. The teeth were then dried, and a buccal-lingual section at the center of the FA was performed using a 0.3 diamond disc (Tizkavan, Tehran, Iran). The sectioned teeth were scrutinized under a stereomicroscope (SKT series stereo microscope, Meiji Techno, Saitama, Japan) at ×20 magnification to assess marginal DP (Figures 2A-2C).

For each molar, the perforation depth from the coronal level of the repair material to the apical end of the perforation and the linear DP between MTA and perforation wall by pretrained single investigator (Y.A.T) was measured in millimeters using vernier caliper (DEMM Calibri, Faenza, Italy), as the extracted molars exhibited slight variation in the distance of the furcation area. Therefore, statistical analysis was conducted to ensure that this distance was not a variable factor influencing the study's results [14,16,17].

Statistical analysis

The collected results were statistically analyzed using the IBM SPSS Statistics for Windows, Version 13 (Released 2005; IBM Corp., Armonk, New York, United States). Initially, a Kolmogorov-Smirnov and Shapiro-Wilk tests indicated normal distribution of DP test in mm among the three groups. One-way ANOVA test was employed to determine significant differences in perforation length (in mm). Subsequently, another one-way ANOVA test was conducted to identify significant differences in methylene blue penetration values (in mm) among the three groups. The Bonferroni post-hoc test was then applied to ascertain significant pairwise differences in methylene blue penetration through two-way comparisons between the groups. The chosen level of significance was set at α = 0.05.

## Results

The study sample included 36 mandibular molars with artificial furcation perforations, evenly distributed into three groups based on the irrigating solution applied after MTA repair. No damage was recorded in any of the samples, and the results were as follows:

Comparison of perforation length between groups

Table 1 presents the mean and standard deviation of simulated perforation depth of molars in the three groups. Additionally, the one-way ANOVA indicates no significant differences in perforation depth values (in mm) among the groups in the sample (p-value = 0.860).

**Table 1:** Comparison of perforation depth between groups (in mm) *One-way ANOVA test ANOVA: Analysis of variance

Groups	Number	Mean ± standard deviation	Minimum	Maximum	F-value	*p-value
A	12	1.72 ± 0.37	1.2	2.3	0.155	0.840
B	12	1.67 ± 0.32	1.2	2.1		
C	12	1.75 ± 0.27	1.3	2		

Comparison of that DP values DP between groups

Table 2 illustrates the mean and standard deviation of DP values in the three groups. Furthermore, the one-way ANOVA reveals significant differences in DP values between groups in the studied sample (p-value < 0.01).

**Table 2:** Mean and range of DP values (in mm) between groups in the studied sample *One-way ANOVA test; ^significant difference ANOVA: Analysis of variance

Groups	Number	Mean ± standard deviation	Minimum	Maximum	F-value	*p-value
A	12	0.78 ± 0.61	0.2	1.8	16.53	<0.001^
B	12	1.53 ± 0.51	0.5	2.1		
C	12	0	0	0		

The pairwise comparisons, with Bonferroni test, indicated that DP values in Group B were higher than those in the group C (p < 0.01). Moreover, DP values in Group A were higher than those in Group C (p= 0.028) and lower than those in Group B (p = 0.004). Overall, a statistical ranking concludes that DP values was obtained as follows (p < 0.05): no Irrigation < NaOCl < CHX.

## Discussion

This study was one of the first to assess the effect of irrigants (NaOCl and CHX) on the sealing ability of hardened MTA margins used to treat furcal perforations. Through clinical observation, it is evident that after applying MTA and sealing the perforation, subsequent root canal treatment is necessary, involving canal preparation and disinfection. This study addresses the impact of accompanying irrigation during root canal treatment on MTA in the pulpal chamber floor.

This study is significant due to the paucity of research examining the effect of various irrigants on MTA in the context of root canal perforation treatments in terms of marginal leakage. ProRoot MTA was chosen as it promotes cementoblast activity, encouraging matrix production for cementum formation, and is biocompatible with periradicular tissues, offering excellent sealing capability when used for perforation repair. Moreover, numerous studies have evaluated the efficacy of different materials in repairing root perforations, with ProRoot MTA demonstrating less leakage compared to amalgam, Portland cement, and IRM. However, the main drawbacks of using MTA for perforation repair are the potential for crown discoloration and its extended setting time, which limits the possibility of completing the treatment in a single visit [1,6,18].

The impact of NaOCl and CHX has been extensively investigated. These irrigants, widely employed in dental practice, are renowned for their efficacy in dissolving organic debris, eliminating residual materials from instrumentation, eradicating pathogenic microorganisms, and debriding the dentin surface [15].

The 5.25% NaOCl and 2% CHX irrigants have been approved for clinical use in managing furcation perforations in permanent molars [19]. However, regarding the irrigation time, no clear protocol was found in the previous literature for irrigating teeth with furcation perforations. Therefore, the irrigation time for CHX (3 min) was based on a previous study that focused on treating necrotic teeth in a single-visit treatment [20], while the irrigation time for NaOCl (10 min) was set to be moderate-not excessive-yet effective against *Enterococcus faecalis* [21]. Moreover, both irrigation times were selected based on laboratory studies similar to the concept of the current study [15,16].

The length of the furcation region is not consistent among teeth. Therefore, the perforation depths were not standardized [16]. However, the statistical analysis in the current study showed no significant difference between groups in terms of furcation depth. This indicates that the perforation depths were homogeneous among the three groups.

DOMs have been widely used in endodontic treatment and offer many advantages: they improve the dentist’s operating position and provide a clear vision of the surgical field, adjustable multiple magnifications, and accuracy in operations [22]. Utilizing a DOM offers enhanced magnification, facilitating the identification of calcified canals, detection of root canal perforations, recognition of isthmuses, interpretation of root canal system complexities, the revelation of microfractures, and aiding in the removal of fractured instruments [23]. Moreover, a magnification of 20x was chosen to observe the entire interface between MTA and dentin in the FA, as higher magnifications did not allow for a full view of the working field.

In this study, the MTA marginal leakage was assessed using the DP method, widely employed in laboratory studies due to its simplicity, nontoxic nature, detectability at low concentrations, and cost-effectiveness compared to alternative techniques, addressing the challenges posed by other available methods [14]. Aqueous methylene blue dye offers several advantages, including its easy penetration into the water compartment of the tooth, nonreactivity with the hard tissues of the tooth, and its detectability under visible light [16]. However, this technique has several drawbacks, including subjectivity in measurement, lack of realistic simulation of clinical conditions, variable dye characteristics, inability to quantify leakage, and limited depth penetration. These limitations can be addressed by using standardized assessment criteria; adopting consistent protocols for dye concentration, immersion time, and sample preparation to improve result reliability across studies; and using dyes with smaller molecular sizes, as was done in this study [24]. Nevertheless, it is still used in many recent studies conducted in Syria [25] and third-world countries [12] due to its relatively low cost and ease of use.

As mentioned by a previous systematic review, bond strength and microleakage are not correlated [26]. However, the limited number of previous studies investigating the effect of irrigants on the marginal leakage of MTA used for repairing furcation perforations necessitated a comparison with similar studies employing different measurement methods like shear bond strength.

The current results indicated significant differences in DP values between groups (p < 0.001). The first null hypothesis was rejected, where MTA was affected by NaOCl and CHX. In this study, there were significant differences in MTA marginal leakage between the no irrigation group (Control Group) and the 5.25% NaOCl group, indicating that NaOCl affects the sealing ability of MTA. A study by Sadegh et al. [10] showed that the control group had the highest bond strength and showed statistically significant differences with NaOCl groups, similar to the current findings. Similarly, Nagas et al. [11] showed that MTA treated with 5.25% NaOCl had lower push-out bond strength than the control group. Moreover, Hong et al. [27] showed that non-accelerated MTA in contact with 2.5% NaOCl had a lower push-out bond strength value than the control group, aligning with our findings.

A possible explanation for the 5% NaOCl effect would be the dissolution of the outermost surface layer of MTA [28]. Accordingly, a significant reduction in the dentine surface microhardness was observed by Butt et al. [29], where the microhardness of white mineral trioxide aggregate (WMTA) and dentin was measured before and after exposure to various solvents. However, according to Uyanik et al. [2], there were no differences between the control sample and NaOCl, and the lowest rate of marginal leakage tested by fluid conductance was achieved with only the NaOCl irrigant group [2]. The variance in findings was rationalized by variations in the assessment approach. While the Uyanik et al. [2] study employed the fluid transport model, the current study utilized the DP method.

Kassab et al. [3] showed that with WMTA repair material, the mean bond strength was not significantly different between irrigant groups. This difference might be justified as evaluation method differences, the differences in methodology including the kind and concentrations of irrigants, and the type and setting time of the tested materials can explain the inconsistency between the studies.

In a recent study similar to the current one, Mohammed et al. [12] found, after evaluating DP values, that NaOCl affects the sealing ability of MTA, especially during its initial setting stages. However, they did not specify the concentration of NaOCl used or the commercial brand of MTA, making it difficult to compare with the results of the current study.

In this study, there were significant differences between MTA wit the no irrigation group and the 2% CHX group, indicating that CHX affects the sealing ability of MTA. A study by Sadegh et al. [10] showed that the control group had the highest bond strength and showed statistically significant differences with CHX groups. This was in line with our findings and showed that 2% CHX significantly decreased the push-out bond strength of MTA to dentin. Hong et al. [27] demonstrated that CHX had detrimental effects on the physical properties and hydration behavior of MTA. Hong et al. [27] showed that the crystals on the CHX-treated surface samples had different chemical distributions, and they were composed of calcium, oxygen, carbon, and silicon, which proved the absence of calcium hydroxide crystals. According to scanning electron microscopic examinations, CHX changed the MTA surface morphology, displaying signs of erosion. These observations may elucidate the significant reduction in the MTA push-out bond strength caused by CHX [30].

In this study, the DP values in the NaOCl group were lower than in the CHX group in the studied sample. Consistent with our results, the Hong et al. [27] study showed that NaOCl-treated groups exhibited significantly higher push-out strength compared to the CHX-treated groups. In the study by Sadegh et al. [10], they revealed no statistically significant differences in the MTA push-out bond strength to dentin between the NaOCl and CHX. This difference in results was justified due to differences in the evaluation method.

The clinical implication drawn from this clinical study is that caution should be exercised when resuming endodontic treatment after the application of MTA in the FA. The use of CHX and NaOCl as irrigants may affect the marginal leakage of MTA.

The limitation of this study lies in the limitations of the DP method itself. Different assessment methods that do not necessitate sectioning are required. Moreover, the current results cannot be compared with other studies regarding the sealing ability of MTA influenced by irrigants due to the limited number of studies. Additionally, the small sample size and the uneven perforation length are limitations. Future in vivo and in vitro studies on different repair materials, such as novel bioceramics, and irrigants with different study methods should be conducted.

## Conclusions

In light of the limitations outlined in this study, we note that it provided detailed and accurate information on the methodology regarding the impact of irrigants on the hardened MTA marginal leakage under standardized laboratory conditions as much as possible. The study found that the sealing capability of MTA was notably influenced by the tested irrigants (CHX and NaOCl), with MTA demonstrating reduced marginal leakage when exposed to both NaOCl and CHX.

It is thus suggested that after applying MTA to seal furcation perforations, sodium hypochlorite can be cautiously used, while CHX should be avoided for root canal irrigation. Alternatively, temporary sealing materials could be used to allow the completion of root canal irrigation and obturation, followed by the removal of the temporary material and final placement of MTA, to ensure optimal sealing of the hardened MTA.

## Figures and Tables

**Figure 3:**
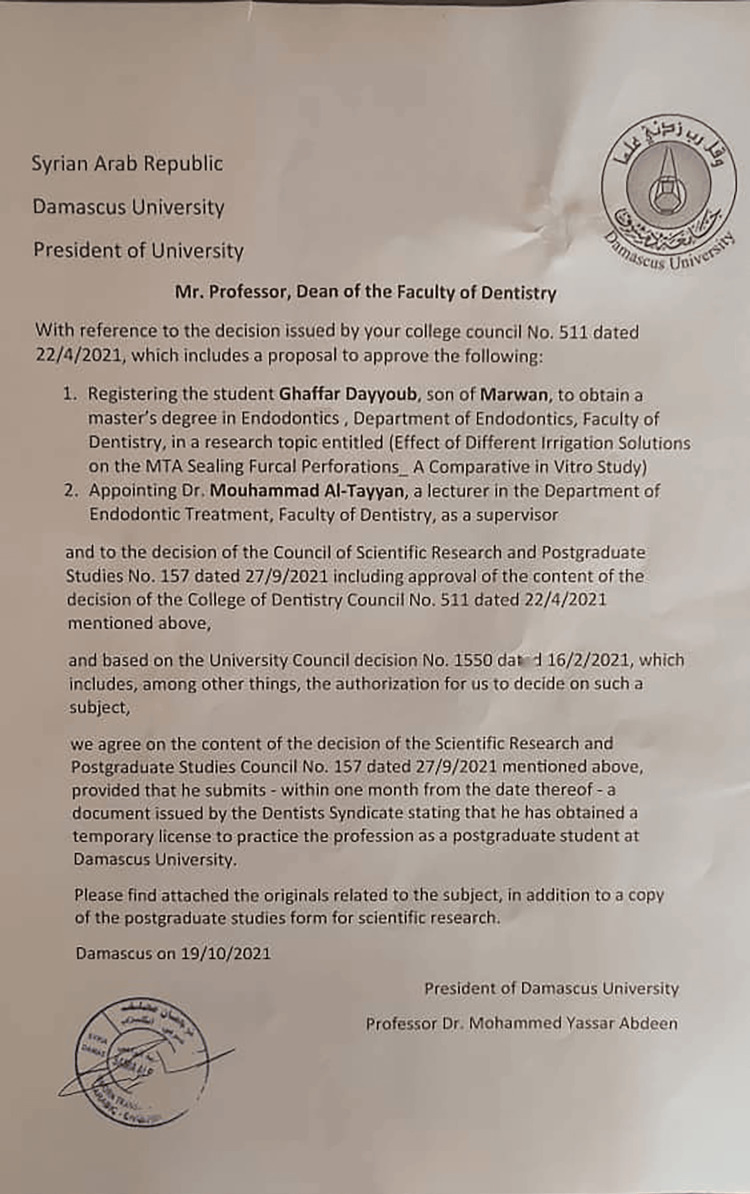
A copy of the IRB approval letter

## Appendices

This in vitro study was approved by the Ethics Committee of Damascus University, Faculty of Dentistry (IRB No. 157 in 27/09/2021). A copy of the IRB approval letter was presented in appendix Figure 3.
